# A Low Advanced Lung Cancer Inflammation Index Predicts a Poor Prognosis in Patients With Metastatic Non–Small Cell Lung Cancer

**DOI:** 10.3389/fmolb.2021.784667

**Published:** 2022-01-14

**Authors:** Ping Lu, Yifei Ma, Jindan Kai, Jun Wang, Zhucheng Yin, Hongli Xu, Xinying Li, Xin Liang, Shaozhong Wei, Xinjun Liang

**Affiliations:** ^1^ Department of Medical Oncology, Hubei Cancer Hospital, Wuhan, China; ^2^ Department of Gastrointestinal Oncology Surgery, Hubei Cancer Hospital, Wuhan, China; ^3^ Department of Thoracic Surgery, Hubei Cancer Hospital, Wuhan, China; ^4^ Department of Epidemiology and Biostatistics, The Ministry of Education Key Lab of Environment and Health, School of Public Health, Huazhong University of Science and Technology, Wuhan, China

**Keywords:** advanced lung cancer inflammation index, inflammation, prognosis, non–small cell lung cancer, overall survival

## Abstract

**Introduction:** Inflammation plays a crucial role in cancers, and the advanced lung cancer inflammation index (ALI) is considered to be a potential factor reflecting systemic inflammation.

**Objectives:** This work aimed to explore the prognostic value of the ALI in metastatic non–small cell lung cancer (NSCLC) and classify patients according to risk and prognosis.

**Methods:** We screened 318 patients who were diagnosed with stage IV NSCLC in Hubei Cancer Hospital from July 2012 to December 2013. The formula for ALI is body mass index (BMI, kg/m^2^) × serum albumin (Alb, g/dl)/neutrophil–lymphocyte ratio (NLR). Categorical variables were analyzed by the chi-square test or Fisher’s exact test. The overall survival (OS) rates were analyzed by the Kaplan–Meier method and plotted with the R language. A multivariate Cox proportional hazard model was used to analyze the relationship between ALI and OS.

**Results:** According to the optimal cut-off value determined by X-tile software, patients were divided into two groups (the ALI <32.6 and ALI ≥32.6 groups), and the median OS times were 19.23 and 39.97 months, respectively (*p* < 0.01). A multivariable Cox regression model confirmed that ALI and chemotherapy were independent prognostic factors for OS in patients with NSCLC. OS in the high ALI group was better than that in the low ALI group (HR: 1.39; 95% CI: 1.03–1.89; *p* = 0.03).

**Conclusions:** Patients with a low ALI tend to have lower OS among those with metastatic NSCLC, and the ALI can serve as an effective prognostic factor for NSCLC patients.

## Introduction

Due to the lack of symptoms in the early stages of lung cancer, only 21% of patients are diagnosed when they are at stage I and 61% of them at advanced stages of lung cancer (Molina et al., 2008; Miller et al., 2019) Because early disease is typically asymp-tomatic, the majority of lung cancers (61%) are diagnosed at stage III or IV; only 21% of cases are diagnosed at stage I. In terms of prognosis, the 5-year survival rate for stage I lung cancer patients is 57%, while that for stage IV patients is significantly lower at 4%. The 5-year relative survival rate for non–small cell lung cancer (NSCLC) patients is 23%, while the 5-year relative survival rate for small-cell lung cancer (SCLC) patients is even lower at 6% (Miller et al., 2019). NSCLC is one of the main causes of cancer-related deaths, and the prognosis of patients with NSCLC is extremely poor. The 5-year overall survival rate of patients with NSCLC at stage IV was less than 5% over the past 10 years (Arbour and Riely, 2019).

Precision medicine is committed to identifying and classifying individual patients to make the best treatment decisions (Vargas and Harris, 2016). Many demographic characteristics and clinicopathological indicators are recognized as prognostic factors for NSCLC patients, and the pathological stage of the tumor is a vital predictor of overall survival (OS). Various combinations of T (primary tumor), N (regional lymph nodes), and M (distant metastasis classification) stages distinguish cancer patients with different survival characteristics (Eberhardt et al., 2015). It has also been confirmed that some demographic characteristics are of great value in predicting the survival time of NSCLC; these include sex, age (Wang et al., 2019), chronic obstructive pulmonary disease (COPD) status (Loganathan et al., 2006; Ytterstad et al., 2016), and smoking status (de Groot and Munden, 2012). Various inflammatory factors, such as the Glasgow prognostic score (GPS) (Sandfeld-Paulsen et al., 2019; Imai et al., 2021), systemic immune-inflammation index (SII) (Tong et al., 2017; Zheng et al., 2021), NLR (Diem et al., 2017; Bongiovanni et al., 2021), and Aarhus composite biomarker score (ACBS) (Sandfeld-Paulsen et al., 2019), have been validated as prognostic markers in lung cancer.

Chronic inflammation can be triggered by the tumor microenvironment (Balkwill and Mantovani, 2001) and plays a vital role in the occurrence, development, and escape of tumors (Perwez Hussain and Harris, 2007). This may be mediated by the excessive secretion of proinflammatory cytokines and other immunosuppressive factors, resulting in damage to DNA (Ikwegbue et al., 2019) and crosstalk in signal transduction pathways. In addition, the susceptibility and severity of cancer may be related to inflammatory cytokines, and the development of cancer is inhibited when inflammatory cytokine expression is lacking or suppressed (Balkwill and Mantovani, 2001). Moreover, inflammation can contribute to cancer-related clinical symptoms, such as anorexia, cachexia, and pain, which seriously affect the quality of life of patients (Batista et al., 2012). There is growing evidence that inflammatory markers can predict the prognosis of patients with various cancers, such as lung cancer (Sarraf et al., 2009), liver cancer (Aleksandrova et al., 2014), and colorectal cancer (Al-Shaer, 2004). Jafri and his colleagues found that the advanced lung cancer inflammation index (ALI), an inflammatory index, can evaluate inflammation and predict survival time in patients with advanced NSCLC and that low ALI is considered to be a risk factor for poor OS (Jafri et al., 2013). ALI is a powerful prognostic biomarker for both NSCLC (Jafri et al., 2013) and SCLC (He et al., 2015) patients. It has been confirmed that low ALI is also associated with a poor prognosis in patients with esophageal cancer (Feng et al., 2014; Tan et al., 2021), diffuse large B-cell lymphoma (Park et al., 2017), HPV-negative head and neck squamous cell carcinoma (Gaudioso et al., 2021), melanoma (Cheng et al., 2021), and colorectal cancer (Pian et al., 2021). Our study aims to evaluate the prognostic value of ALI in patients with metastatic NSCLC. The results are consistent with Jafri and colleagues’ finding that the ALI can be used as a valuable prognostic indicator for NSCLC patients.

## Materials and Methods

### Study Design

The study is a cross-sectional survey of cancer patients, a total of 318 of whom were pathologically diagnosed with stage IV NSCLC at Hubei Cancer Hospital (HBCH) between July 2012 and December 2013. We selected patients on the basis of the following inclusion criteria: 1) age >18 years, 2) pathological diagnosis of NSCLC, and 3) metastatic pathologic stage IV according to the American Joint Committee on Cancer (AJCC) Staging Manual (Seventh Edition). The exclusion criteria were as follows: 1) second primary cancer at NSCLC diagnosis, 2) a history of malignancy or hematologic disease, 3) blood test results and clinical symptoms and signs indicating severe infection status, and 4) missing follow-up data. Of the 351 eligible patients, we excluded 33 based on missing data on variables of interest. Finally, 318 patients were analyzed further.

### Demographic and Clinical Variables

Related inflammatory indicators, including serum albumin (Alb), neutrophil count, lymphocyte count, and lactate dehydrogenase (LDH) were collected. Furthermore, demographic baseline and clinicopathological characteristics, including age, gender, smoking and drinking status, cancer location, family history, treatment of cancer, and history of lung-related diseases, were obtained through medical records. Body mass index (BMI) was derived using its established derivation formula: body weight (kg)/height squared (m^2^). The neutrophil–lymphocyte ratio (NLR) was calculated as follows: peripheral blood absolute neutrophil count divided by absolute lymphocyte count. The formula for the ALI was BMI ×Alb/NLR, where the unit of BMI is kg/m (Molina et al., 2008), and the unit of Alb is g/dl.

### Follow-Up

In this study, we defined OS as the period spanning from the date of pathological diagnosis of NSCLC to the date of the final follow-up (i.e., December 31, 2013) or the date of censoring the patient as alive or dead. The follow-up started from the diagnosis in Hubei Cancer Hospital in December 2013 and continued until the end of the follow-up period or the loss of follow-up. During this period, patients underwent routine reexaminations, such as blood laboratory tests and imaging tests.

### Statistical Analysis

The optimal cut-off values of ALI and LDH were determined through X-tile and used to convert these factors into categorical variables. The chi-square test and Fisher’s exact test were used to analyze the relationships among the categorical variables. The OS rate was analyzed by the Kaplan–Meier method, and the survival differences were assessed for statistical significance using the log-rank test. The median survival time and 95% confidence interval (CI) were reported for each group. Furthermore, survival curves including 95% CIs were generated using R language. The influence of variables on OS was analyzed by multivariate Cox proportional hazard regression, and variables that reached statistical significance (*p* < 0.05) and were associated with ALI were included in the multivariable analysis. Moreover, the hazard ratio (HR) was estimated. All tests were bilateral, and *p* < 0.05 was considered the threshold for statistical significance. Statistical analyses were performed by SPSS 25.0 software.

## Results

### Baseline Characteristics

The demographic and clinical variables of the patients who were pathologically diagnosed with NSCLC are shown in Table 1. The majority of patients (n = 221) were younger than 65 years old, and 66.4% of patients were male. A history of cigarette smoking and alcohol consumption was reported by 164 (51.6%) and 78 (24.5%) patients, respectively. In total, 16 (5%) patients had a history of COPD, and 18 (5.7%) had a history of tuberculosis. Regarding treatment, 73% (n = 232) of patients accepted chemotherapy, and 12.6% of patients (n = 40) were treated with radiotherapy. The level of LDH in 74.5% of the patients was lower than 274.4, and the ALI level of most patients was low, ALI <32.6. The median survival time and 95% confidence interval of patients in different groups were obtained by univariate survival analysis. Among them, patients who received chemotherapy tended to have a longer survival time (*p* = 0.003). The difference in survival time between patients with different levels of LDH and ALI was considered to be statistically significant; furthermore, patients with low levels of LDH (*p* = 0.013) and a high ALI (*p* = 0.003) had longer survival times.

**TABLE 1:** Baseline characteristics and median OS.

Variable	N (%)	Median OS, Months (95% CI)	P
Age			
<65	221 (69.5)	30.60 (21.16–40.04)	0.23
≥65	97 (30.5)	20.93 (12.78–29.10)	—
Gender			
Male	211 (66.4)	22.27 (13.50–31.04)	0.47
Female	107 (33.6)	32.40 (21.60–43.20)	—
Smoking Status			
Never	154 (48.4)	28.13 (19.90–36.38)	0.95
Current or ever	164 (51.6)	22.27 (12.1–32.35)	—
Drinking Status			
Never	240 (75.5)	26.37 (18.00–34.73)	0.80
Current or ever	78 (24.5)	26.2 (12.93–39.50)	—
Location			
Left	186 (58.5)	28.83 (18.98–38.70)	0.38
Right	132 (41.5)	21.00 (11.74–30.13)	—
Family history of cancer			
Yes	60 (81.1)	29.4 (17.09–41.71)	0.40
No	258 (18.9)	25.23 (17.11–33.36)	—
COPD			
Yes	16 (5)	16.90 (3.64–30.16)	0.35
No	302 (95)	26.37 (18.94–33.79)	—
Tuberculosis			
Yes	18 (5.7)	14.67 (7.46–21.87)	0.44
No	300 (94.3)	26.97 (19.95–33.99)	—
Chemotherapy			
Yes	232 (73)	32.40 (25.61–39.20)	0.003
No	86 (27)	14.83 (9.96–19.71)	—
Radiotherapy			
Yes	40 (12.6)	18.4 (11.90–24.91)	0.23
No	278 (87.4)	28.13 (19.66–36.61)	—
LDH			
<274.4	237 (74.5)	31.53 (24.75–38.32)	0.013
≥274.4	81 (25.5)	16.60 (12.81–20.39)	—
ALI			
<32.6	191 (60.0)	19.23 (13.39–25.09)	0.003
≥32.6	127 (40.0)	39.97 (33.51–46.43)	—

### Relationship Between Baseline Characteristic Variables and Advanced Lung Cancer Inflammation Index Analysed by Chi-Square Test or Fisher’s Exact Test

According to X-tile software, the optimal cut-off value of ALI was determined to be 32.6. Then, all patients were divided into two groups: ALI<32.6 (n = 191) and ALI ≥32.6 (n = 127). The optimal cut-off value of LDH was determined to be 274.4. The relationship between demographic and clinical variables and ALI was analyzed by the chi-square test or Fisher’s exact test, as shown in Table 2. The results indicated that gender (*p* = 0.045) and LDH (*p* < 0.001) were significantly associated with ALI.

**TABLE 2:** Basic characteristics according to the level of ALI.

Variable	ALI <32.6	ALI ≥32.6	P
Age			
≥65	134 (70.2)	87 (68.5)	0.75
≥65	57 (29.8)	40 (31.5)	—
Gender			
Male	135 (70.7)	76 (59.8)	0.045
Female	56 (29.3)	51 (40.2)	—
Smoking Status			
Never	90 (47.1)	64 (50.4)	0.57
Current or ever	101 (52.9)	63 (49.6)	—
Drinking Status			
Never	147 (77.0)	93 (73.2)	0.45
Current or ever	44 (23.0)	34 (26.8)	—
Location			
Left	118 (61.8)	68 (53.5)	0.14
Right	73 (38.2)	59 (46.5)	—
Family history of cancer			
Yes	37 (19.4)	23 (18.1)	0.78
No	154 (80.6)	104 (81.9)	—
COPD			
Yes	8 (4.2)	8 (6.3)	0.40
No	183 (95.8)	119 (93.7)	—
Tuberculosis			
Yes	14 (7.3)	4 (3.1)	0.11
No	177 (92.7)	123 (96.9)	—
Chemotherapy			
Yes	132 (69.1)	100 (78.7)	0.06
No	59 (30.9)	27 (21.3)	—
Radiotherapy			
Yes	25 (13.1)	15 (11.8)	0.74
No	166 (86.9)	112 (88.2)	—
LDH			
<274.4	127 (66.5)	110 (86.6)	<0.001
≥274.4	64 (33.5)	17 (13.4)	—

### Univariate Survival Analysis and Survival Curves

**FIGURE 1:**
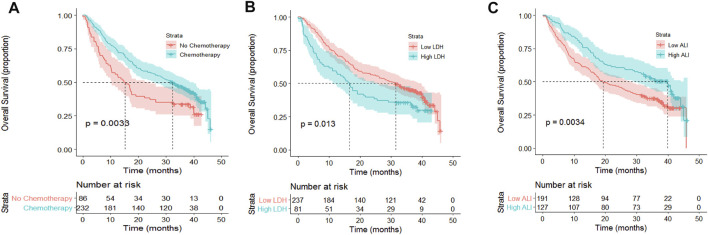
Kaplan–Meier curves for OS and risk tables according to chemotherapy, LDH, and ALI level. **(A)** OS stratification according to chemotherapy. No chemotherapy was significantly associated with poor OS (*p* = 0.003). **(B)** OS stratification according to LDH. High LDH was significantly associated with poor OS (*p* = 0.013). **(C)** OS stratification according to ALI. Low ALI was significantly associated with poor OS (*p* = 0.003).

Some factors were recognized as associated with poor OS according to the results of univariate survival analysis (Figure 1), including no chemotherapy (*p* = 0.003; Figure A), high LDH (*p* = 0.013; Figure B), and low ALI (*p* = 0.003; Figure C). According to the Kaplan–Meier survival curve, the median survival times in the no chemotherapy and chemotherapy groups were 14.83 months (95% CI: 9.96–19.71 months) and 32.40 months (95% CI: 25.61–39.20 months), respectively, signifying a marked difference, as revealed by the log-rank test (*p* = 0.003). Moreover, the high LDH group had a shorter median OS period than the low LDH group (16.60 vs 31.53 months, *p* = 0.013). The OS of patients with a high ALI (≥32.6) was longer than that of patients with a low ALI (<32.6) (39.97 vs 19.23 months, respectively).

### Multivariate Cox Regression Model

A multivariate Cox proportional hazard model was used to analyze the influence of variables on OS and estimated its HR with 95% CI. Gender, chemotherapy, LDH, and ALI were included in the multivariate Cox regression model (Table 3). Chemotherapy (*p* = 0.01) and ALI (*p* = 0.03) were independent prognostic factors in terms of OS. Furthermore, the risk of death in patients with low ALI was 1.39 times higher than that in patients with high ALI (HR: 1.39; 95% CI: 1.03–1.89; *p* = 0.03).

**TABLE 3:** Multivariable Cox regression model (adjusted for gender, chemotherapy, LDH, and ALI).

Variables	Hazard Ratio	95% CI	p
Gender			
Male	—	—	—
Female	0.90	0.67–1.22	0.50
Chemotherapy			
No	—	—	—
Yes	0.67	0.49–0.91	0.01
LDH			
<274.4	—	—	—
≥274.4	1.33	0.96–1.83	0.08
ALI			
≥32.6	—	—	—
<32.6	1.39	1.03–1.89	0.03

## Discussion

Inflammation is an automatic defense response against pathogens, and inflammatory cytokines contribute to reactive oxygen species production, DNA damage, cell proliferation, and tumor-related angiogenesis in the tumor microenvironment. Inflammation contributes to the occurrence, development, and immune escape of tumors and even affects the treatment response.

The NLR and C-reactive protein (CRP) level have been proven to be prognostic factors for NSCLC and other tumors, including hepatocellular carcinoma (Liao et al., 2018), colorectal cancer (Tsai et al., 2016), and esophageal cancer (Otowa et al., 2019). It has been reported that patients with NSCLC whose NLR returned to normal after one cycle of systematic treatment had a better prognosis than those whose NLR was still not in the normal range (Cedrés et al., 2012). However, in a study of advanced renal cell carcinoma, it was found that the remission rate of NLR after treatment was not related to the survival rate (Keizman et al., 2012). An increase in neutrophil count or a decrease in lymphocyte count can lead to an increase in the NLR. Neutrophils can produce cytokines and inhibit lymphocyte-mediated immune activity, thus affecting the prognosis of tumor patients.

Another study confirmed that hypoalbuminemia is related to a poor prognosis in NSCLC patients who are treated with erlotinib (Fiala et al., 2016). The correlation between hypoalbuminemia and shorter survival after tumor resection is statistically significant and has been confirmed in resectable colon cancer. It is well known that Alb is one of the indicators for assessing nutritional status. In addition, albumin is an acute phase protein that can indicate inflammatory activity; it can bind to other laboratory indicators, such as C-reactive protein, lymphocytes, and globulins, and its predictive value has been evaluated.

Moreover, as a nutritional status assessment indicator, BMI is also associated with the prognosis of cancer patients. Both underweight and morbidly obese statuses are associated with poor survival in NSCLC and SCLC (Shepshelovich et al., 2019). Similarly, Masaaki et al. explained that both low BMI and high BMI are related to an increased risk of poor survival in breast cancer (Kawai et al., 2012). For thyroid cancer, patients who have a high BMI might have a higher risk of suffering from cancer (Son et al., 2018; Abdel-Rahman, 2019).

As a metric reflecting BMI, Alb, and NLR, ALI provides a more comprehensive assessment of inflammation than these indicators alone. A low ALI value means higher systemic inflammatory activity and plays an important role in the prognosis of patients. A high ALI suggests low activity systemic inflammation in cancer patients, which may result from moderately increased BMI, increased albumin, and decreased NLR. These factors can be involved in the inhibition of tumor occurrence, invasion, and metastasis, promoting a good prognosis. Conversely, a low ALI is usually associated with a poor prognosis. In the univariate survival analysis, there was a significant correlation between a low ALI and poor OS (*p* = 0.003). In the multivariate Cox regression analysis, we adjusted for gender, chemotherapy, and LDH, and the results proved that a low ALI is an independent risk factor for OS in NSCLC patients. (HR = 1.39; *p* = 0.03). Therefore, we proposed that ALI can serve as an effective prognostic factor for NSCLC patients.

## Conclusion

Our study confirms that the difference in survival time of metastatic NSCLC patients with different ALI statuses is statistically significant, and tumor patients with a low ALI have lower OS. Due to the clinical feasibility of assessing the ALI, it can be used to help distinguish patients with different prognoses.

## Data Availability

The datasets used and/or analyzed during this study are available from the corresponding authors upon reasonable request.

## References

[B1] Abdel-Rahman O. (2019). Prediagnostic BMI and Thyroid Cancer Incidence in the PLCO Trial. Future Oncol. 15 (30), 3451–3456. 10.2217/fon-2019-0292 31646903

[B2] Al-Shaer M. H. (2004). C-reactive Protein and Risk of colon Cancer. JAMA 291 (23), 2819. author reply 2819. 10.1001/jama.291.23.2819-a 15199030

[B3] Aleksandrova K. Boeing H. Nöthlings U. Jenab M. Fedirko V. Kaaks R. (2014). Inflammatory and Metabolic Biomarkers and Risk of Liver and Biliary Tract Cancer. Hepatology 60 (3), 858–871. 10.1002/hep.27016 24443059 PMC4231978

[B4] Arbour K. C. Riely G. J. (2019). Systemic Therapy for Locally Advanced and Metastatic Non-small Cell Lung Cancer: A Review. JAMA 322 (8), 764–774. 10.1001/jama.2019.11058 31454018

[B5] Balkwill F. Mantovani A. (2001). Inflammation and Cancer: Back to Virchow? The Lancet 357 (9255), 539–545. 10.1016/s0140-6736(00)04046-0 11229684

[B6] Batista M. L. Jr. Peres S. B. McDonald M. E. Alcantara P. S. M. Olivan M. Otoch J. P. (2012). Adipose Tissue Inflammation and Cancer Cachexia: Possible Role of Nuclear Transcription Factors. Cytokine 57 (1), 9–16. 10.1016/j.cyto.2011.10.008 22099872

[B7] Bongiovanni A. Foca F. Menis J. Stucci S. L. Artioli F. Guadalupi V. (2021). Immune Checkpoint Inhibitors with or without Bone-Targeted Therapy in NSCLC Patients with Bone Metastases and Prognostic Significance of Neutrophil-To-Lymphocyte Ratio. Front. Immunol. 12, 697298. 10.3389/fimmu.2021.697298 34858389 PMC8631508

[B8] Cedrés S. Torrejon D. Martínez A. Martinez P. Navarro A. Zamora E. (2012). Neutrophil to Lymphocyte Ratio (NLR) as an Indicator of Poor Prognosis in Stage IV Non-small Cell Lung Cancer. Clin. Transl Oncol. 14 (11), 864–869. 10.1007/s12094-012-0872-5 22855161

[B9] Cheng X. Dong Y. Lou F. (2021). The Predictive Significance of the Advanced Lung Cancer Inflammation Index (ALI) in Patients with Melanoma Treated with Immunotherapy as Second-Line Therapy. Cmar Vol. 13, 173–180. 10.2147/cmar.s286453 PMC7810587 33469361

[B10] de Groot P. Munden R. F. (2012). Lung Cancer Epidemiology, Risk Factors, and Prevention. Radiologic Clin. North America 50 (5), 863–876. 10.1016/j.rcl.2012.06.006 22974775

[B11] Diem S. Schmid S. Krapf M. Flatz L. Born D. Jochum W. (2017). Neutrophil-to-Lymphocyte Ratio (NLR) and Platelet-To-Lymphocyte Ratio (PLR) as Prognostic Markers in Patients with Non-small Cell Lung Cancer (NSCLC) Treated with Nivolumab. Lung Cancer 111, 176–181. 10.1016/j.lungcan.2017.07.024 28838390

[B12] Eberhardt W. E. Mitchell A. Crowley J. Kondo H. Kim Y. T. Turrisi A. (2015). The IASLC Lung Cancer Staging Project: Proposals for the Revision of the M Descriptors in the Forthcoming Eighth Edition of the TNM Classification of Lung Cancer. J. Thorac. Oncol. 10 (11), 1515–1522. 10.1097/JTO.0000000000000673 26536193

[B13] Feng J. Huang Y. Chen Q. (2014). A New Inflammation index Is Useful for Patients with Esophageal Squamous Cell Carcinoma. Ott 7, 1811–1815. 10.2147/ott.s68084 PMC4199817 25336972

[B14] Fiala O. Pesek M. Finek J. Racek J. Minarik M. Benesova L. (2016). Serum Albumin Is a strong Predictor of Survival in Patients with Advanced-Stage Non-small Cell Lung Cancer Treated with Erlotinib. Neoplasma 63 (3), 471–476. 10.4149/318_151001N512 26952513

[B15] Gaudioso P. Borsetto D. Tirelli G. Tofanelli M. Cragnolini F. Menegaldo A. (2021). Advanced Lung Cancer Inflammation index and its Prognostic Value in HPV-Negative Head and Neck Squamous Cell Carcinoma: a Multicentre Study. Support Care Cancer 29 (8), 4683–4691. 10.1007/s00520-020-05979-9 33515105 PMC8236476

[B16] He X. Zhou T. Yang Y. Hong S. Zhan J. Hu Z. (2015). Advanced Lung Cancer Inflammation Index, a New Prognostic Score, Predicts Outcome in Patients with Small-Cell Lung Cancer. Clin. Lung Cancer 16 (6), e165–e171. 10.1016/j.cllc.2015.03.005 25922292

[B17] Ikwegbue P. C. Masamba P. Mbatha L. S. Oyinloye B. E. Kappo A. P. (2019). Interplay between Heat Shock Proteins, Inflammation and Cancer: a Potential Cancer Therapeutic Target. Am. J. Cancer Res. 9 (2), 242–249. 30906626 PMC6405974

[B18] Imai H. Kishikawa T. Minemura H. Yamada Y. Ibe T. Yamaguchi O. (2021). Pretreatment Glasgow Prognostic Score Predicts Survival Among Patients with High PD‐L1 Expression Administered First‐line Pembrolizumab Monotherapy for Non‐small Cell Lung Cancer. Cancer Med. 10 (20), 6971–6984. 10.1002/cam4.4220 34414673 PMC8525165

[B19] Jafri S. H. Shi R. Mills G. (2013). Advance Lung Cancer Inflammation index (ALI) at Diagnosis Is a Prognostic Marker in Patients with Metastatic Non-small Cell Lung Cancer (NSCLC): a Retrospective Review. BMC cancer 13, 158. 10.1186/1471-2407-13-158 23530866 PMC3618002

[B20] Kawai M. Minami Y. Nishino Y. Fukamachi K. Ohuchi N. Kakugawa Y. (2012). Body Mass index and Survival after Breast Cancer Diagnosis in Japanese Women. BMC Cancer 12, 149. 10.1186/1471-2407-12-149 22510365 PMC3444378

[B21] Keizman D. Ish-Shalom M. Huang P. Eisenberger M. A. Pili R. Hammers H. (2012). The Association of Pre-treatment Neutrophil to Lymphocyte Ratio with Response Rate, Progression Free Survival and Overall Survival of Patients Treated with Sunitinib for Metastatic Renal Cell Carcinoma. Eur. J. Cancer 48 (2), 202–208. 10.1016/j.ejca.2011.09.001 22018713 PMC3483077

[B22] Liao M. Chen P. Liao Y. Li J. Yao W. Sun T. (2018). Preoperative High-Sensitivity C-Reactive Protein to Lymphocyte Ratio index Plays a Vital Role in the Prognosis of Hepatocellular Carcinoma after Surgical Resection. Ott Vol. 11, 5591–5600. 10.2147/ott.s167857 PMC6135434 30237725

[B23] Loganathan R. S. Stover D. E. Shi W. Venkatraman E. (2006). Prevalence of COPD in Women Compared to Men Around the Time of Diagnosis of Primary Lung Cancer. Chest 129 (5), 1305–1312. 10.1378/chest.129.5.1305 16685023

[B24] Miller K. D. Nogueira L. Mariotto A. B. Rowland J. H. Yabroff K. R. Alfano C. M. (2019). Cancer Treatment and Survivorship Statistics, 2019. CA A. Cancer J. Clin. 69 (5), 363–385. 10.3322/caac.21565 31184787

[B25] Molina J. R. Yang P. Cassivi S. D. Schild S. E. Adjei A. A. (2008). Non-small Cell Lung Cancer: Epidemiology, Risk Factors, Treatment, and Survivorship. Mayo Clinic Proc. 83 (5), 584–594. 10.1016/s0025-6196(11)60735-0 PMC2718421 18452692

[B26] Otowa Y. Nakamura T. Yamazaki Y. Takiguchi G. Nakagawa A. Yamamoto M. (2019). Meaning of C-Reactive Protein Around Esophagectomy for cStage III Esophageal Cancer. Surg. Today 49 (1), 90–95. 10.1007/s00595-018-1706-z 30167922

[B27] Park Y. H. Yi H. G. Lee M. H. Kim C. S. Lim J. H. (2017). Prognostic Value of the Pretreatment Advanced Lung Cancer Inflammation Index (ALI) in Diffuse Large B Cell Lymphoma Patients Treated with R-CHOP Chemotherapy. Acta Haematol. 137 (2), 76–85. 10.1159/000452991 28076862

[B28] Perwez Hussain S. Harris C. C. (2007). Inflammation and Cancer: an Ancient Link with Novel Potentials. Int. J. Cancer 121 (11), 2373–2380. 10.1002/ijc.23173 17893866

[B29] Pian G. Hong S. Y. Oh S. Y. (2021). Prognostic Value of Advanced Lung Cancer Inflammation index in Patients with Colorectal Cancer Liver Metastases Undergoing Surgery. Tumori, 300891620983465. 10.1177/0300891620983465 33393453

[B30] Sandfeld-Paulsen B. Meldgaard P. Sorensen B. S. Safwat A. Aggerholm-Pedersen N. (2019). The Prognostic Role of Inflammation-Scores on Overall Survival in Lung Cancer Patients. Acta Oncologica 58 (3), 371–376. 10.1080/0284186x.2018.1546057 30632850

[B31] Sarraf K. M. Belcher E. Raevsky E. Nicholson A. G. Goldstraw P. Lim E. (2009). Neutrophil/lymphocyte Ratio and its Association with Survival after Complete Resection in Non-small Cell Lung Cancer. J. Thorac. Cardiovasc. Surg. 137 (2), 425–428. 10.1016/j.jtcvs.2008.05.046 19185164

[B32] Shepshelovich D. Xu W. Lu L. Fares A. Yang P. Christiani D. (2019). Body Mass Index (BMI), BMI Change, and Overall Survival in Patients with SCLC and NSCLC: A Pooled Analysis of the International Lung Cancer Consortium. J. Thorac. Oncol. 14 (9), 1594–1607. 10.1016/j.jtho.2019.05.031 31163278 PMC6734935

[B33] Son H. Lee H. Kang K. Lee I. (2018). The Risk of Thyroid Cancer and Obesity: A Nationwide Population-Based Study Using the Korea National Health Insurance Corporation Cohort Database. Surg. Oncol. 27 (2), 166–171. 10.1016/j.suronc.2018.03.001 29937167

[B34] Tan X. Peng H. Gu P. Chen M. Wang Y. (2021). Prognostic Significance of the L3 Skeletal Muscle Index and Advanced Lung Cancer Inflammation Index in Elderly Patients with Esophageal Cancer. Cmar Vol. 13, 3133–3143. 10.2147/cmar.s304996 PMC8043796 33859499

[B35] Tong Y.-S. Tan J. Zhou X.-L. Song Y.-Q. Song Y.-J. (2017). Systemic Immune-Inflammation index Predicting Chemoradiation Resistance and Poor Outcome in Patients with Stage III Non-small Cell Lung Cancer. J. Transl Med. 15 (1), 221. 10.1186/s12967-017-1326-1 29089030 PMC5664920

[B36] Tsai P. L. Su W. J. Leung W. H. Lai C. T. Liu C. K. (2016). Neutrophil-lymphocyte Ratio and CEA Level as Prognostic and Predictive Factors in Colorectal Cancer: A Systematic Review and Meta-Analysis. J. Cancer Res. Ther. 12 (2), 582–589. 10.4103/0973-1482.144356 27461614

[B37] Vargas A. J. Harris C. C. (2016). Biomarker Development in the Precision Medicine Era: Lung Cancer as a Case Study. Nat. Rev. Cancer 16 (8), 525–537. 10.1038/nrc.2016.56 27388699 PMC6662593

[B38] Wang H. Hou J. Zhang G. Zhang M. Li P. Yan X. (2019). Clinical Characteristics and Prognostic Analysis of Multiple Primary Malignant Neoplasms in Patients with Lung Cancer. Cancer Gene Ther. 26 (11-12), 419–426. 10.1038/s41417-019-0084-z 30700800

[B39] Ytterstad E. Moe P. Hjalmarsen A. (2016). COPD in Primary Lung Cancer Patients: Prevalence and Mortality. Int. J. Chron. Obstruct Pulmon Dis. 11, 625–636. 10.2147/copd.s101183 27042050 PMC4809346

[B40] Zheng K. Liu X. Ji W. Lu J. Cui J. Li W. (2021). The Efficacy of Different Inflammatory Markers for the Prognosis of Patients with Malignant Tumors. Jir Vol. 14, 5769–5785. 10.2147/jir.s334941 PMC8573157 34764670

